# Metal‐HisTag coordination for remote loading of very small quantities of biomacromolecules into PLGA microspheres

**DOI:** 10.1002/btm2.10272

**Published:** 2022-02-17

**Authors:** Jason Albert, Rae Sung Chang, George A. Garcia, Steven P. Schwendeman

**Affiliations:** ^1^ Department of Pharmaceutical Sciences and the Biointerfaces Institute University of Michigan Ann Arbor Michigan USA; ^2^ Department of Medicinal Chemistry University of Michigan Ann Arbor Michigan USA; ^3^ Department of Biomedical Engineering University of Michigan Ann Arbor Michigan USA; ^4^ Present address: PTC Therapeutics, 311 Pennington Rocky Hill Rd., Pennington NJ USA

**Keywords:** biologics, drug delivery, drug discovery, metal coordination, PLGA, remote loading

## Abstract

Challenges to discovery and preclinical development of long‐acting release systems for protein therapeutics include protein instability, use of organic solvents during encapsulation, specialized equipment and personnel, and high costs of proteins. We sought to overcome these issues by combining remote‐loading self‐healing encapsulation with binding HisTag protein to transition metal ions. Porous, drug‐free self‐healing microspheres of copolymers of lactic and glycolic acids with high molecular weight dextran sulfate and immobilized divalent transition metal (M^2+^) ions were placed in the presence of proteins with or without HisTags to bind the protein in the pores of the polymer before healing the surface pores with modest temperature. Using human serum albumin, insulin‐like growth factor 1, and granulocyte‐macrophage colony‐stimulating factor (GM‐CSF), encapsulated efficiencies of immunoreactive protein relative to nonencapsulation protein solutions increased from ~41%, ~23%, and ~9%, respectively, without Zn^2+^ and HisTags to ~100%, ~83%, and ~75% with Zn^2+^ and HisTags. These three proteins were continuously released in immunoreactive form over seven to ten weeks to 73%–100% complete release, and GM‐CSF showed bioactivity >95% relative to immunoreactive protein throughout the release interval. Increased encapsulation efficiencies were also found with other divalent transition metals ions (Co^2+^, Cu^2+^, Ni^2+^, and Zn^2+^), but not with Ca^2+^. Ethylenediaminetetraacetic acid was found to interfere with this process, reverting encapsulation efficiency back to Zn^2+^‐free levels. These results indicate that M^2+^‐immobilized self‐healing microspheres can be prepared for simple and efficient encapsulation by simple mixing in aqueous solutions. These formulations provide slow and continuous release of immunoreactive proteins of diverse types by using a amount of protein (e.g., <10 μg), which may be highly useful in the discovery and early preclinical development phase of new protein active pharmaceutical ingredients, allowing for improved translation to further development of potent proteins for local delivery.

## 1 INTRODUCTION

Over the last several decades, the landscape of pharmaceutical drug products has been transformed from a near monolith of small molecules to a diverse space with biologics gaining more and more dominance. In 1982, the first genetically engineered form of insulin was approved.^1^ By 2017, half of the top 10 best‐selling drug products over the previous 15 years were biologics.^2^ Recombinant proteins, fusion proteins, antibodies, and others biologics have led to therapeutic breakthroughs in a number of treatment areas. They are also costlier and often more complicated to discover, develop, formulate, and manufacture. Another challenge of biologics is that they must be injected, rather than taken orally like most small‐molecule drug products, which is a significant impediment to patient compliance.^3^ To reduce the number of injections and increase patient compliance, controlled‐release formulations have been developed, which require weekly, biweekly, or monthly injections rather than daily injections for noncontrolled‐release formulations. Particularly useful for proteins, controlled release can also be helpful for local delivery to hard‐to‐reach areas, like the brain,^4^, ^5^ joints,^6^, ^7^ and posterior segment of the eye.^8^, ^9^ One difficulty, however, in the evaluation and development of new protein APIs, which require slow release to evaluate drug efficacy, is that large quantities of the biomacromolecule are generally required during formulation of controlled release dosage forms. Moreover, common encapsulation procedures require trained personnel with the use of organic solvent‐based unit operations.^3^ These combined factors can significantly impede the early drug development process when producing and using large amounts of the proteins of interest can be financially infeasible.^10^ Here, we aim to address these cases by creating a simple, general, and low‐cost paradigm for preparation of local controlled‐release dosage forms for protein drug discovery that could be simple enough for most any bench scientist to use.

Copolymers of lactic (or lactide) and glycolic (or glycolide) acids (PLGAs) have become a desired delivery vehicle for a wide variety of therapeutics, including peptides, proteins, antibodies, vaccine antigens, and nucleic acids. Advantages of PLGA microspheres include biocompatibility and biodegradability, injectability of PLGA microspheres through a syringe needle with minimal discomfort, and tunable and long‐term complete release of the therapeutics, including peptides and proteins.^11^, ^12^, ^13^, ^14^, ^15^ PLGA is used in at least 19 FDA‐approved controlled‐release products on the market in the US,^3^, ^16^, ^17^ and therefore usually is the first biodegradable polymer considered for such applications.

While many hurdles of PLGA drug product formulation have been overcome, one long‐standing issue is protein stability during encapsulation.^18^ Traditional methods of encapsulation in PLGA microspheres require exposing biomacromolecules to micronization, organic/aqueous interfaces, air/water interfaces, high shear stress, organic solvents, and high temperatures, all of which can result in instability or aggregation of the biomacromolecule and low encapsulation efficiencies.^16^, ^19^, ^20^, ^21^, ^22^, ^23^, ^24^, ^25^

To avoid these stressors and the resulting damage to protein and low encapsulation efficiency, our group previously devised organic solvent‐free self‐healing microencapsulation, in which porous PLGA microspheres are mixed with an aqueous solution of biomacromolecule.^22^ The temperature is then raised above the PLGA glass transition temperature (*T*_*g*_), causing the pores in the surface of the microspheres to heal, encapsulating the biomacromolecule within the microspheres.^26^, ^27^ In active remote loading, a trapping agent is contained in the drug‐free self‐healing microspheres before exposure to the biomacromolecule to dramatically increase encapsulation efficiency.^18^, ^28^ The charge interaction between cationic peptides and the negatively charged carboxylic end‐group of PLGA chains has also been targeted as an active remote loading strategy for smaller net cationic peptides that do not require preservation of tertiary structure.^29^ Using active remote loading, encapsulation efficiencies greater than 95% have been achieved with elevated drug loading (>7% wt/wt).^18^, ^28^ Examples of trapping agents include aluminum‐ and calcium‐based adjuvants^22^, ^30^, ^31^, ^32^, ^33^ for vaccines and glycosaminoglycan‐like biopolymers^18^ that often bind growth factors. One drawback of these methods is that the trapping agent must be paired with specific biomacromolecules that have binding affinity for said trapping agent, and therefore, the methods are not universal. Here, we aim to take advantage of the coordination binding between divalent transition metals and poly‐histidine tags (HisTags) to create an active remote loading method that is more applicable to a broader spectrum of recombinant proteins.

Immobilized metal affinity chromatography (IMAC) was first developed in 1975 as a method of separating and purifying proteins based on their cysteine and histidine content.^34^ These amino acids form coordination bonds with transition metals like Ni^2+^, Cu^2+^, and Zn^2+^.^35^ Thus, proteins rich in cysteine and histidine can be purified by flowing them through a column with immobilized divalent transition metals. As the capability to express recombinant proteins expanded, so did strategies for IMAC. Today, HisTags (typically His_6_ or His_10_) can be expressed at the C‐ or N‐terminus end of peptides and proteins, allowing them to be easily purified via IMAC.^36^ To elute purified proteins or peptides of interest from the column, the pH can be lowered to protonate the histidine, interrupting the coordination bond, or imidazole, glycine, or a chelating agent like ethylenediaminetetraacetic acid (EDTA) can be added to the column buffer solution, displacing the molecule of interest.^37^

**FIGURE 1:**
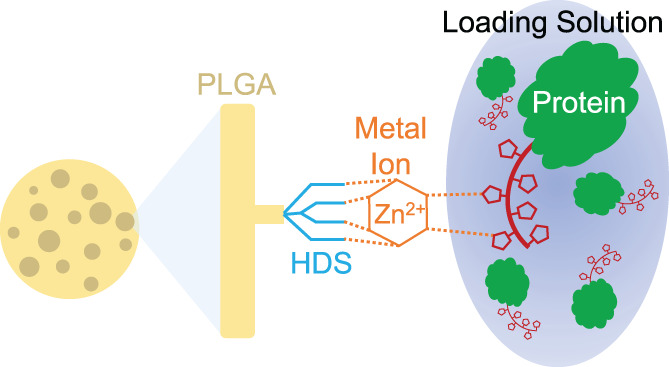
Schematic of remote loading mechanism into porous PLGA microspheres as related to immobilized metal affinity chromatography (IMAC). PLGA acts as the support structure. HDS acts as the chelating agent, immobilizing the metal ion, which binds a HisTag protein out of the loading solution through the porous network within the microsphere

In the active remote loading and self‐encapsulation platform described in Figure 1, our approach is to directly encapsulate high‐molecular‐weight dextran sulfate (HDS), a negatively charged branched polysaccharide,^18^ in drug‐free and porous PLGA microspheres to serve as a metal‐immobilizing scaffold. A divalent metal cation is then bound to the HDS to serve as a trapping agent for HisTag proteins before self‐healing encapsulation. Our goal is to create a remote‐loading controlled‐release platform that (1) is virtually universal for any recombinant peptide or protein; (2) is highly efficient; (3) uses very small quantities of the protein or peptide; (4) slowly and continuously releases active protein; and (5) can be performed by scientists without training in microencapsulation and without specialized mixing or drying equipment. Meeting these goals would make this platform attractive during the discovery and early development of biologics, when producing and using large amounts of the molecules of interest could be very costly or infeasible and controlled‐release efficacy data are desired. Due to the low quantities encapsulated here, local delivery of potent proteins is the targeted application of this platform. For example, a single injection of only 0.01% protein‐loaded PLGA implants for controlled release of basic fibroblast growth factor was sufficient to rescue limbs and restore perfusion in a murine hindlimb ischemic model.^38^

For decades, unpredictable or inadequate pharmacokinetics, resulting in undesirable toxicology and poor efficacy, has plagued drug candidates in clinical trials.^39^ To combat this, drug delivery and formulation scientists have been introduced earlier in the development process. Allowing drug researchers with or without formulation expertise to easily and cost‐effectively test early‐stage drug candidates with a controlled‐release formulation in vivo could allow for much better translation from the bench to the patient.^40^ We offer this platform as a potential solution to this critical translational need.

## 2 EXPERIMENTAL METHODS

### 2.1 Materials

Resomer RG 504 PLGA (50:50, ester‐terminated, molecular weight 38,000–54,000 Da), magnesium carbonate, trehalose, 88% hydrolyzed poly(vinyl alcohol) (PVA), and high‐molecular‐weight (>500,000 Da) dextran sulfate (HDS) were purchased from Sigma Aldrich. Zinc, copper, cobalt, nickel, and calcium acetate salts were purchased from Sigma Aldrich. Poly‐histidine tagged (HisTag) Human Serum Albumin (HSA) was purchased from Arco Biosystems and untagged (NoTag) HSA was purchased from Raybiotech. HisTag and NoTag granulocyte‐macrophage colony‐stimulating factor (GM‐CSF) was purchased from Sino Biological. HisTag and NoTag insulin‐like growth factor 1 (IGF‐1) was purchased from Signalway Antibodies. EDTA and bovine serum albumin (BSA) were purchased from Sigma Aldrich. Blocker casein in phosphate buffered saline (PBS) was purchased from Thermo Fisher Scientific. All other common reagents and solvents were purchased from Sigma Aldrich, except where otherwise specified.

### 2.2 Preparation of microspheres

Porous PLGA microspheres with HDS as a metal immobilizer, MgCO_3_ as a pH‐modulator and porosigen,^18^, ^19^ and trehalose as a porosigen were prepared by double water–oil–water (w/o/w) emulsion and solvent evaporation. The first emulsion was created by homogenizing 1 ml of 250 mg/ml PLGA and 6% wt/wt MgCO_3_ in methylene chloride with an inner‐water phase of 200 μl of 4% wt/vol HDS and 3% wt/vol trehalose in a glass cell culture tube at 18,000 rpm for 60 s over an ice bath, using the Tempest IQ^2^. The second emulsion was created by adding 2 ml of 5% PVA to the primary emulsion and vortexing for 60 s. The w/o/w double emulsion was added to 100 ml of 0.5% PVA and stirred for 3 h at room temperature in a 150‐ml beaker to allow for hardening and evaporation of methylene chloride. The 20–60 μm fraction of microspheres was collected using sieves and the microspheres were washed with double‐distilled water and lyophilized.

### 2.3 Assessment of microsphere morphology by scanning electron microscopy

The surface morphology of microspheres was examined via a Tescan MIRA3 FEG electron microscope (SEM). Microspheres were mounted onto a brass stub via double‐sided adhesive tape and sputtered with gold for 60 s at 40 W under vacuum. Images were taken at an excitation voltage of 5 kV. Prior to imaging, microspheres were incubated in protein‐free loading solution for the specified duration at the specified temperature rotating at 30 rpm, then washed with double‐distilled water and lyophilized.

### 2.4 Remote loading and encapsulation of metals and proteins

#### 2.4.1 Standard Procedure

Metals were remotely loaded into the PLGA microspheres by incubating the microspheres in at least 1 ml of 500 mM metal acetate salt solution (or water as a control) per 1 mg of microspheres for 24 h rotating at 30 rpm at room temperature. Microspheres were washed with double‐distilled water under vacuum on a 0.2 μm nylon filter and lyophilized. Remote loading HisTag and NoTag protein solutions were prepared by buffer exchange with Amicon ultra centrifugal filter units (for HSA and 10 μg/ml GM‐CSF) or by diluting lyophilized powders in loading solution. Proteins were remotely loaded into the metal‐loaded microspheres by incubating 1 mg of microspheres in 100 μl of 50 μg/ml protein (HisTag or NoTag), unless otherwise specified at 10 μg/ml GM‐CSF in one case, in 50 mM sodium acetate, 300 mM sodium chloride, pH 8.0 solution (loading solution) for 48 h at room temperature rotating at 30 rpm followed by 42 h at 43°C rotating at 30 rpm to induce healing and pore‐closure.

#### 2.4.2 Inhibition of remote loading with EDTA

The effect of EDTA on the capacity of Zn‐loaded microspheres to remotely load HisTag HSA was determined by incubating Zn‐loaded microspheres (or water‐incubated microspheres as a control) in 50% saturated EDTA solution (or water as a control) for 24 h rotating at 30 rpm at room temperature. Microspheres then underwent HSA loading as described above and loading and encapsulation were determined using Coomassie Plus protein assay as described below.

#### 2.4.3 Effect of divalent metal cation on remote loading

To examine the effect of different divalent metal cations on the remote loading and encapsulation of HisTag IGF‐1, zinc acetate, copper acetate, cobalt acetate, nickel acetate, or calcium acetate were used in the metal loading step as described above. Metal loading percentage and IGF‐1 encapsulation efficiency were quantified as described below.

### 2.5 Determination of divalent metal cation loaded

The amount of divalent metal cation remotely loaded into microspheres was determined by dissolving several mg of microspheres in acetone, centrifuging for 5 min at 8000 rpm, and removing the supernatant for three cycles. The pellet was then reconstituted in water and analyzed using a Perkin‐Elmer Nexion 2000 ICP‐MS using appropriate standards and scandium as an internal standard. Metal cation loading percentage was calculated as (mass of metal cation in microspheres/total mass of microspheres) × 100.

### 2.6 Determination of immunoreactive protein by ELISA

HSA and IGF‐1 ELISA kits were purchased from Raybiotech and performed according to kit instructions to determine immunoreactive protein concentrations. GM‐CSF ELISA kits were purchased from Raybiotech and PeproTech and were similarly applied. In all ELISAs, NoTag and HisTag proteins used for remote loading encapsulation were also included as reference standards.

### 2.7 Determination of total protein by Coomassie Plus protein assay

Total protein content for No Tag and HisTag HSA in loading solutions was measured by Coomassie Plus protein assay using a 1:1 sample‐to‐reagent ratio. BSA standards were used, with the HSA proteins included as reference standards, and absorbance was read at 595 nm in accordance with the protocol.

### 2.8 Estimation of protein loading and encapsulation efficiency

Protein loading (*l*) in microspheres was estimated by ELISA and Coomassie Plus protein assay by comparing the final concentrations of protein in the loading solution to a control loading solution, which underwent the same conditions without microspheres as follows:

$$l=V\left(C_{C}-C_{\mathrm{MS}}\right),$$

where *V* (=0.1 ml), *C*_*C*_, and *C*_MS_ are the volume of loading solution, concentration of protein in control loading solution, and the concentration of protein in the loading solution with microspheres, respectively.

Encapsulation efficiency of the available active protein (i.e., relative to unencapsulation control) was calculated as:

$${\mathrm{EE}}_{\text{avail}}=\frac{C_{C}-C_{\mathrm{MS}}}{C_{C}}\times 100\%,$$

where *C*_*C*_ and *C*_MS_ are the concentration of protein in control loading solution and the concentration of protein in the loading solution with microspheres quantified by ELISA, respectively. Encapsulation efficiency of available total protein was calculated similarly with concentrations of protein quantified by Coomassie Plus protein assay used in place of those measured by ELISA.

Actual active encapsulation efficiency of active protein was calculated by:

$${\mathrm{EE}}_{\text{actual}}=\frac{C_{C}-C_{\mathrm{MS}}}{C_{i}}\times 100\%,$$

where *C*_*C*_ *, C*_MS_, and *C*_*i*_ are the concentration of protein in control loading solution, concentration of protein in the loading solution with microspheres, and the original concentration of protein in the loading solution quantified by ELISA, respectively. Actual total encapsulation efficiency was calculated similarly with concentrations of protein quantified by Coomassie Plus protein assay used in place of those measured by ELISA.

### 2.9 Evaluation of release kinetics

HSA release was conducted by incubating 1 mg microspheres in 1 ml PBS + 0.02% Tween 80 + 1% casein, pH 7.4. IGF‐1 release was conducted from 1 mg microspheres in 1 ml PBS + 0.02% Tween 80 + 1% BSA, pH 7.4. GM‐CSF release was conducted from 1 mg microspheres in 1 ml PBS + 0.02% Tween 80 + 1% BSA, pH 7.4 or 1 ml 0.1 M HEPES buffer +1% BSA, pH 7.4. Media was completely replaced at each timepoint. All samples were incubated at 37°C with shaking. Casein was used as a blocking agent in place of BSA for HSA release to avoid interference in the HSA ELISA. HEPES was used in place of PBS in one instance in an effort to measure Zn^2+^ release, as phosphate salts are known to co‐precipitate Zn.^41^

### 2.10 GM‐CSF activity assay

The activity of HisTag GM‐CSF released from Zn‐loaded microspheres was determined using the PathHunter® Sargramostim Bioassay Kit from Eurofins DiscoverX. HisTag GM‐CSF was included as a reference standard.

### 2.11 Statistics

All significance testing was conducted using one‐tailed Student's *t* tests. Statistical significance was considered *p* < 0.5.

## 3 RESULTS AND DISCUSSION

To test our approach, HDS and MgCO_3_ were co‐encapsulated in the porous PLGA 50/50 microspheres, as described previously.^18^ These microspheres were originally designed to microencapsulate growth factors that are known to bind to extracellular matrix. In these formulations, HDS binds the growth factor and MgCO_3_ is present to both inhibit acid drop caused by PLGA hydrolysis and provide continuous release by production of salt when reacting to low‐molecular‐weight degradation products.^18^, ^42^ We demonstrated high loading and encapsulation efficiency, and slow release of vascular endothelial growth factor without significant loss of immunoreactivity or heparin‐binding affinity for weeks during slow and continuous release. Basic proteins, bFGF20 and lysozyme, were similarly encapsulated.^18^ To expand the capability of these microspheres to deliver a wider spectrum of proteins, we bound Zn^2+^ and other divalent metal cations to HDS/PLGA microspheres by incubating 1 ml of acetate salt solution of the cation in the presence of a modest 1 mg of microspheres at room temperature for 24 h before loading the protein. ICP‐MS showed that Zn^2+^ was significantly loaded into the microspheres after Zn‐acetate exposure at a level of 0.42 ± 0.12% wt/wt%.

**FIGURE 2:**
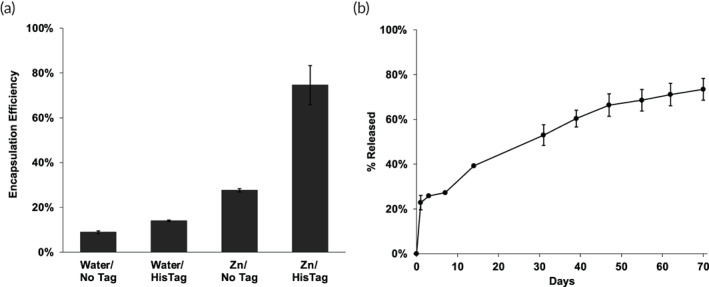
HisTag GM‐CSF is efficiently encapsulated in Zn^2+^‐immobilized PLGA microspheres by remote loading and slowly released. (a) Active available protein encapsulation efficiency of NoTag and HisTag GM‐CSF into Zn^2+^‐free and Zn^2+^‐immobilized PLGA microspheres from ~10 μg/ml protein loading solution. (b) Release of immunoreactive HisTag GM‐CSF from Zn^2+^‐immobilized PLGA microspheres in 1 ml PBS + 0.02% Tween 80 + 1% BSA, pH 7.4 at 37°C. One μg protein and 1 mg of microspheres in 100 μl loading solution for self‐healing encapsulation. Zn/HisTag *EE_avail_* significantly greater than each control; *p* < 0.05

After metal‐ion uptake in HDS/PLGA microspheres, exchanging the solution to a 100 μl solution of 1 μg of HisTag granulocyte‐macrophage colony‐stimulating factor (HisTag GM‐CSF), which has local delivery applications,^43^, ^44^, ^45^ and raising the temp for 42 h at 43°C resulted in an estimated self‐healing microencapsulation of ~75% protein available in the loading solution (Figure 2a; Table 1). If the Zn^2+^ was not added before loading, the encapsulation efficiency (EE) dropped to ~14%. If GM‐CSF was added without a HisTag, the EE was ~28% for Zn^2+^/HDS/PLGA and ~ 9% for HDS/PLGA. The release kinetics of the resulting HisTag GM‐CSF in the self‐healed Zn^2+^/HDS/PLGA microspheres is shown in Figure 2b. After a modest initial burst release, a continuous release of protein was recorded by ELISA over 70 days. Because of the focus on protein drug discovery, we did not seek to further stabilize the encapsulated protein and/or examine the immunoreactivity of any protein remaining in the polymer after the release incubation. Scanning electron micrographs also confirmed that open surface pores were maintained until the final heated self‐healing step (Figure S1). Hence, this proof‐of‐principle experiment shows that a protein that does not seem to bind well to HDS can be encapsulated on a very small scale with Zn^2+^/HDS/PLGA microspheres when using the HisTag version and then slowly release immunoreactive protein under physiological conditions for months. Note that the SEM images were acquired after washing and drying the microspheres. Therefore, the polymer loses the swollen state that exists during incubation and the drying creates an altered morphology under the electron microscope. However, the number and size of the pores on the dry microsphere surface in the micrographs when evaluated at each stage of the aqueous encapsulation procedure is useful to confirm the healing of the polymer, as we have demonstrated in previous studies.^18^, ^22^, ^26^, ^27^

**TABLE 1:** Summary of remote self‐healing encapsulation by Zn^2+^‐HisTag protein binding (EE, encapsulation efficiency)

Protein	Active protein loaded (μg/mg)	Total protein loaded (μg/mg)	Active available EE	Total available EE	Actual active EE	Actual total EE
GM‐CSF (~10 μg/ml)	0.21 ± 0.03	–	75 ± 9%	–	37 ± 4%	–
GM‐CSF (50 μg/ml)	1.9 ± 0.3	2.3 ± 0.2	55 ± 7%	49 ± 1%	37 ± 6%	46 ± 3%
IGF‐1 (50 μg/ml)	2.4 ± 0.9	–	81 ± 23%	–	48 ± 17%	–
HSA (50 μg/ml)	3.5 ± 0.2	4.6 ± 0.4	100 ± 3%	97 ± 2%	70 ± 2%	92 ± 6%

**FIGURE 3:**
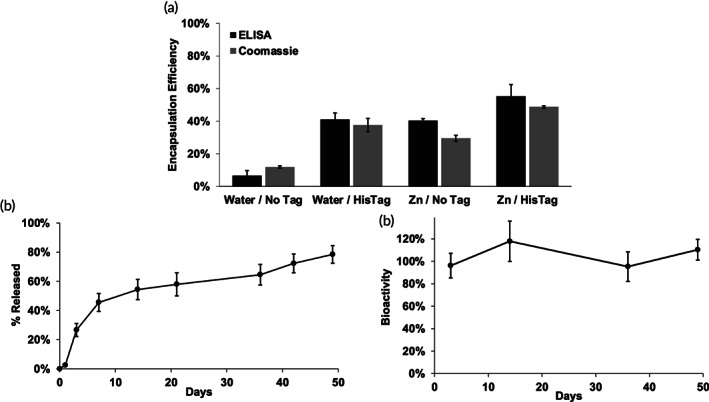
HisTag GM‐CSF is efficiently encapsulated in Zn^2+^‐immobilized PLGA microspheres by remote loading and slowly released while maintaining bioactivity. (a) Active and total available protein encapsulation efficiency of NoTag and HisTag GM‐CSF into Zn^2+^‐free and Zn^2+^‐immobilized PLGA microspheres from 50 μg/ml protein loading solution. (b) Release of immunoreactive HisTag GM‐CSF from Zn^2+^‐immobilized PLGA microspheres in 0.1 M HEPES + 1% BSA, pH 7.4 at 37°C. (c) Bioactivity of released GM‐CSF relative to immunoreactive protein. Five micrograms protein and 1 mg of microspheres in 100 μl loading solution were used for self‐healing encapsulation. Zn/HisTag *EE_avail_* by ELISA and total protein assay significantly greater than each control; *p* < 0.05

Although promising, the above remote loading example used a very low concentration of protein and only 49.5 ± 1% of protein remained immunoreactive in the control solution. Proteins at these low concentrations commonly bind to vessel walls even if coated with low‐protein‐binding materials.^46^ We then increased the protein concentration in the loading media to 50 μg/ml HisTag GM‐CSF. In this case, the loading efficiency decreased slightly to ~55%, and the differential advantage relative to the Zn^2+^‐free/HisTag or Zn^2+^/NoTag controls also decreased slightly (Figure 3a). Moreover, encapsulation of active and total protein by employing ELISA (55 ± 7%) and Coomassie Plus protein assay (49 ± 1%), respectively, were performed and shown to yield consistent values. Here, 68 ± 6% of the protein in the control loading solution remained immunoreactive after incubation at loading conditions, which is much higher than at the lower concentration. Once again, we observed slow and continuous release of immunoreactive HisTag GM‐CSF for 49 days (Figure 3b) The bioactivity of the protein was also monitored according to a CSF2RA‐CSF2RB dimerization cell‐based assay (Figure 3c). As seen in the figure, there was no noticeable loss in bioactivity over the entire release interval.

**FIGURE 4:**
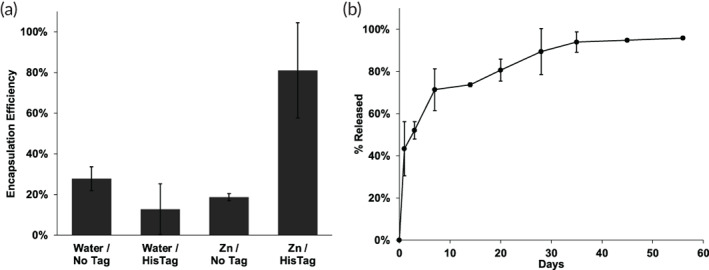
HisTag GM‐CSF is efficiently encapsulated in Zn^2+^‐immobilized PLGA microspheres by remote loading and slowly released while maintaining bioactivity. (a) Active and total available protein encapsulation efficiency of NoTag and HisTag GM‐CSF into Zn^2+^‐free and Zn^2+^‐immobilized PLGA microspheres from 50 μg/ml protein loading solution. (b) Release of immunoreactive HisTag GM‐CSF from Zn^2+^‐immobilized PLGA microspheres in 0.1M HEPES + 1% BSA, pH 7.4 at 37°C. (c) Bioactivity of released GM‐CSF relative to immunoreactive protein. Five micrograms protein and 1 mg of microspheres in 100 μl loading solution were used for self‐healing encapsulation. Zn/HisTag *EE_avail_* by ELISA and total protein assay significantly greater than each control; *p* < 0.05

While promising for GM‐CSF, we further tested our approach with a second protein, IGF‐1, which also has local delivery utility,^47^, ^48^, ^49^, ^50^, ^51^ at the higher 50 μg/ml level. As expected, the HisTag IGF‐1 was loaded in the self‐healing Zn^2+^/HDS/PLGA microspheres at about 80% efficiency, as measured by ELISA (Figure 4a). All other controls displayed encapsulation efficiencies of ~20% or less. Here, 59% ± 13% of the protein in the control loading solution remained immunoreactive after incubation at loading conditions. Release of the protein was again continuous and nearly complete over 56 days, although with a larger burst release than for GM‐CSF (Figure 4b).

**FIGURE 5:**
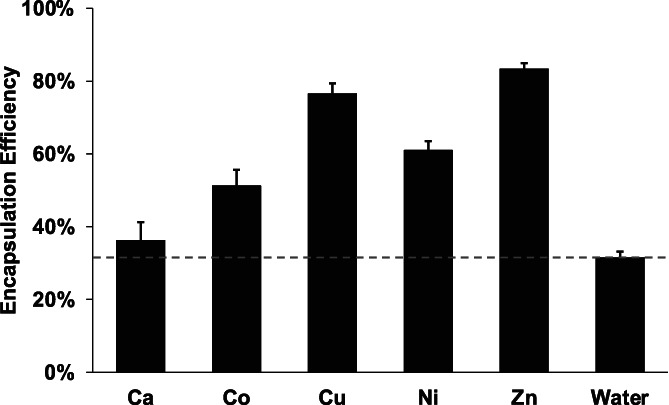
The immobilization of divalent transition metals improves the total protein encapsulation efficiency of HisTag IGF‐1 into PLGA microspheres. Encapsulation efficiency of HisTag IGF‐1 into Ca^2+^‐, Co^2+^‐, Cu^2+^‐, Ni^2+^‐, and Zn^2+^‐immobilized, and M^2+^‐free PLGA microspheres. Ca^2+^ *EE_avail_* by total protein assay not significantly greater than water control; *p* > 0.05. All transition metal *EE_avail_* by total protein assay significantly greater than water and Ca^2+^ controls; *p* <0.05

To examine the effect of the divalent cation on our approach, self‐healing HDS/PLGA microspheres were exposed to acetate salts of transition metals (Co^2+^, Cu^2+^, Ni^2+^, and Zn^2+^), an alkaline earth metal (Ca^2+^), or no salt control. Compared to the no salt control, HisTag IGF‐1 was encapsulated with higher efficiency in the microspheres exposed to divalent transition metals. Accounting for the amount of metal loaded into the microspheres (Figure S2) and the amount of protein encapsulated in the metal‐free microspheres, the encapsulated protein above control per mole of metal ion followed Cu^2+^>Zn^2+^>Ni^2+^>Co^2+^ >>>Ca^2+^, as expected by the relative affinity of these cations for HisTag (Figure 5).^34^ Microspheres exposed to Ca^2+^ showed no substantial difference in EE as compared to the no salt control, as Ca^2+^ is known to possess much less affinity than transition metals for HisTags. Hence, these data further strongly support the HisTag‐to‐transition metal binding occurring during loading of the HisTag protein before self‐healing encapsulation.

**FIGURE 6:**
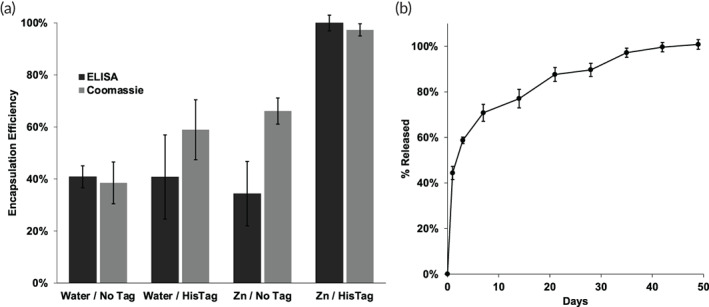
HisTag HSA is efficiently encapsulated in Zn^2+^‐immobilized PLGA microspheres by remote loading and slowly released. (a) Active and total protein encapsulation efficiency of NoTag and HisTag HSA into Zn^2+^‐free and Zn^2+^‐immobilized PLGA microspheres from 50 μg/ml protein loading solution. (b) Release of immunoreactive HisTag HSA from Zn^2+^‐immobilized PLGA microspheres in 1 ml PBS + 0.02% Tween 80 + 1% casein, pH 7.4 at 37°C. Zn/HisTag *EE_avail_* by ELISA and total protein assay significantly greater than each control; *p* < 0.05

We then applied a third protein, HSA, as a model protein to examine various phenomena at lower cost and to further support the generality of the approach. As shown in Figure 6a, again the HisTag HSA bound preferentially to the Zn^2+^/HDS/PLGA microspheres, with an EE of >95%. Controls without HisTag, without Zn^2+^, and without HisTag and Zn^2+^ were higher for HSA than for the previously studied proteins, but nonetheless all below ~41% as measured by ELISA. Here, 70% ± 1% of the protein in the control loading solution remained immunoreactive after incubation at loading conditions. HisTag HSA release from the standard formulation was complete, and slow and continuous after a modest initial burst by ELISA (Figure 6b).

**FIGURE 7:**
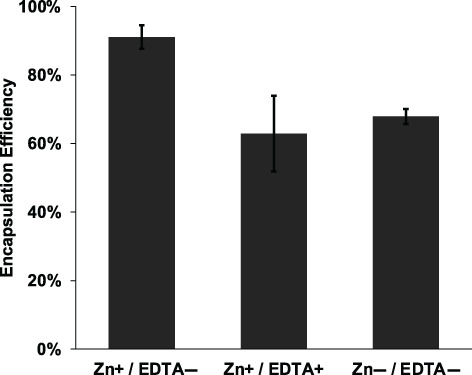
EDTA interferes with the ability of Zn^2+^‐immobilized PLGA microspheres to efficiently encapsulate HisTag HSA. Encapsulation efficiency of HisTag HSA into Zn^2+^‐immobilized PLGA microspheres without and with incubation with EDTA and into Zn^2+^‐free PLGA microspheres without incubation with EDTA from 50 μg/ml loading solution as determined by mass loss from loading solution compared to control loading solution, measured by Coomassie assay. Zn+/EDTA *EE_avail_* by total protein assay significantly greater than other treatments; *p* < 0.05

To further probe the Zn^2+^‐HisTag coordination, we tested whether EDTA, a strong chelating agent often used to elute HisTag proteins of IMAC columns, interfered with the encapsulation of HisTag proteins. To do this, after exposing HDS/PLGA microspheres to Zn^2+^ but before exposing the microspheres to HisTag HSA, we exposed the microspheres to EDTA. The EDTA/Zn^2+^/HDS/PLGA microspheres showed nearly the same EE as microspheres that had been exposed to neither Zn^2+^ nor EDTA, and far lower efficiency than the standard formulation (Figure 7). These data again support the HisTag‐to‐transition metal binding and are consistent with EDTA entirely inhibiting the coordination.

### 3.1 Protein stability considerations

As previously discussed, the stability of proteins is a major obstacle in controlled‐release formulations. Our data strongly support the stable, immunoreactive, and bioactive encapsulation and release of a wide variety of HisTag proteins via Zn^2+^/HDS/PLGA microspheres. This formulation evolved from multiple improvements in protein stabilization during encapsulation and release. The poorly soluble base, MgCO_3_, has been shown capable of helping to obviate pH‐induced protein damage from aliphatic ester‐capped PLGA 50/50 under specific formulation conditions.^52^ The common damage to protein during organic solvent exposure and excess mixing was averted by making use of passive polymer healing to allow encapsulation under aqueous conditions with gentle agitation.^22^ Finally, when combining a protein‐binding excipient that largely remains in the polymer during loading such as HDS, we found that both high efficiency loading of protein drugs and further stabilization during release was observed.^18^

While this encapsulation method avoids many of the harsh stressors of direct double emulsion and solvent evaporation encapsulation, it is not free of potential damage to the protein. The relatively high pH (8) of the loading solution buffer needed to optimize the interaction between the metal cations and the HisTag proteins can be deleterious to proteins, particularly at elevated temperature.^53^, ^54^ The slightly high temperature (43°C) used to heal the microspheres can cause unfolding and/or aggregation of some proteins. Indeed, we did see decreases in immunoreactivity of proteins after exposure to loading conditions (Table 1).

### 3.2 Potential use of metal‐HisTag binding for self‐healing encapsulation during discovery of biologic phase

During biologic drug discovery and early development, the slow and continuous release of immunoreactive and bioactive protein is crucial so that protein candidates can be studied in vivo in hard‐to‐reach areas like the brain, eye, and joints, where repeated injection may not be feasible, or in tissue engineering applications where local growth factor support is also desired.^55^ Aside from PLGA formulations, osmotic pumps are another option, but these can be cumbersome and difficult to apply in certain cases.^39^ For traditional PLGA formulations, though, depending on the desired protein loading, typically more than 1–100 mg of protein is often used (with the lower level often accompanying a second bulk protein excipient such as albumin^38^, ^52^) to formulate microspheres using traditional direct encapsulation batch methods depending on the target loading, and there is no specific binding mechanism used to help stabilize the protein. For these reasons, studying candidates in the proper formulation and pharmacokinetic settings is difficult, leading to costly failures or missed opportunities.^56^, ^57^ Using the method described here, immunoreactive and bioactive protein can be encapsulated and slowly released using just 1–5 μg of protein (in a 100 μl loading solution and 1 mg of microspheres). Others have developed micro‐ and nanoparticle encapsulation methods for biologics that require very small quantities of drug, for example with poly(ethylene‐co‐vinyl acetate),^58^ or with PLGA.^38^, ^52^ However, these methods require organic solvent, are not generalizable, and require specialized equipment and training to perform encapsulation. It is noted that the current goal here is develop a more universal formulation for simple and low‐cost remote loading of proteins in the drug discovery phase and not to identify a final formulation for development. This represents a drug delivery solution to a drug discovery problem, with the aim of improving translation from discovery to preclinical studies and, eventually, to the clinic. Further enhancement of drug stability and release would be the next logical goal once desirable drug candidates for further development were identified.

### 3.3 Limitations and future work

Here we have demonstrated the basic concept to utilize M^2+^‐HisTag binding to remotely load small quantities of proteins for controlled release. One potential limitation of this technique is that the HisTag must not impair the biological activity of the encapsulated protein. It is likely that this can be mitigated by the option of his‐tagging either the amino‐ or carboxy‐termini of the proteins.^36^ Cleavable HisTags are often used in molecular biology^59^ and it is possible that a physiologically cleavable HisTag could be utilized. There are multiple ways in which the approach could be improved and expanded in the future. To improve the stability of proteins during encapsulation, the temperature should be decreased. We have previously shown that the addition of plasticizer to the polymer can cause healing at lower temperatures (e.g., 37°C) as the hydrated *T*_*g*_ of PLGA is lowered.^22^ Plasticization could also reduce the healing time, which further simplify the encapsulation method. Second, the initial burst in some of the above examples is higher than usually desired. We anticipate there are potential ways to mitigate this issue, such as controlling the distribution of M^2+^ in the polymer matrix and the matrix porosity/microstructure. Likewise, replacement of MgCO_3_ with the less soluble base, ZnCO_3_, has been shown to reduce the initial burst of albumin from PLGA.^60^ Third, protein loading will need to be increased in order to expand utility for systemic delivery or for local delivery of much less potent proteins (e.g., monoclonal antibodies). Fourth, it will be important to demonstrate in important animal models the utility of this approach for novel proteins. Due to the very low quantities of protein used in these experiments, direct measurement of loading is difficult. While LECO nitrogen analysis would require larger quantities, it is possible amino acid analysis or other colorimetric or fluorometric digestive assays could be used to directly measure the loaded protein.^61^, ^62^, ^63^ Alternatively, the proteins may be quantified utilizing HisTag specific dyes or antibodies.^64^, ^65^ Finally, HisTags are not the only widely applicable affinity tags. It is not difficult to imagine replacing the HisTag‐metal‐ion pair with a maltose binding protein (MBP)‐maltose pair or a glutathione S‐transferase (GST)‐glutathione pair. These and other affinity tag‐ligand pairs will be important to examine in the future.

## AUTHOR CONTRIBUTIONS

**Jason Albert:** Data curation (lead); formal analysis (lead); funding acquisition (supporting); investigation (equal); methodology (equal). **Rae Sung Chang:** Data curation (supporting); formal analysis (supporting); methodology (equal). **George A. Garcia:** Conceptualization (equal); methodology (supporting).

## CONFLICT OF INTERESTS

The authors declare that there are no conflict of interests.

### PEER REVIEW

The peer review history for this article is available at https://publons.com/publon/10.1002/btm2.10272.

## Supporting information


**Figure S1** A porous network is created, maintained, and healed. (a,b) Scanning electron micrographs of PLGA microspheres prepared as described. (c,d) Scanning electron micrographs of PLGA microspheres prepared without the inclusion of trehalose in the inner‐water phase. (e,f) Scanning electron micrographs of PLGA microspheres following incubation at room temperature for 48 h rotating at 30 rpm. (g,h) Scanning electron micrographs of PLGA microspheres following incubation at room temperature for 48 h rotating at 30 rpm and at 43°C for 42 h rotating at 30 rpm
**Figure S2** Divalent metal cations are remotely loaded into PLGA microspheres via simple mixing. Weight‐by‐weight loading as measured by ICP

## Data Availability

The data that support the findings of this study are available from the corresponding author upon reasonable request.
