# Supplementary material for: Metal‐HisTag coordination for remote loading of very small quantities of biomacromolecules into PLGA microspheres
Source: Bioeng Transl Med. 2022 Feb 17;7(2):e10272. doi: 10.1002/btm2.10272 (PMC9115689; doi:10.1002/btm2.10272)
Supplement: Supplementary file 1 — Figure S1 A porous network is created, maintained, and healed. (a,b) Scanning electron micrographs of PLGA microspheres prepared as described. (c,d) Scanning electron micrographs of PLGA microspheres prepared without the inclusion of trehalose in the inner‐water phase. (e,f) Scanning electron micrographs of PLGA microspheres following incubation at room temperature for 48 h rotating at 30 rpm. (g,h) Scanning electron micrographs of PLGA microspheres following incubation at room temperature for 48 h rotating at 30 rpm and at 43°C for 42 h rotating at 30 rpm Figure S2 Divalent metal cations are remotely loaded into PLGA microspheres via simple mixing. Weight‐by‐weight loading as measured by ICP [file BTM2-7-e10272-s001.docx]

**Appendix I**

**Supporting Information**

**Figure S1.** A porous network is created, maintained, and healed. a,b) Scanning electron micrographs of PLGA microspheres prepared as described. c,d) Scanning electron micrographs of PLGA microspheres prepared without the inclusion of trehalose in the inner-water phase. e,f) Scanning electron micrographs of PLGA microspheres following incubation at room temperature for 48 h rotating at 30 rpm. g,h) Scanning electron micrographs of PLGA microspheres following incubation at room temperature for 48 h rotating at 30 rpm and at 43 °C for 42 h rotating at 30 rpm.

**Figure S2.** Divalent metal cations are remotely loaded into PLGA microspheres via simple mixing. Weight-by-weight loading as measured by ICP.
